# Factors affecting survival of foals with pneumonia in a referral hospital

**DOI:** 10.1186/s12917-024-04405-0

**Published:** 2024-12-18

**Authors:** Heini Sofia Rossi, Heli Katariina Hyytiäinen, Jouni Juho Tapio Junnila, Minna Marjaana Rajamäki, Anna Kristina Mykkänen

**Affiliations:** 1 Department of Equine and Small Animal Medicine, Faculty of Veterinary Medicine, University of Helsinki, Viikintie 49, Helsinki, FI-00014 Finland; 2 EstiMates Oy, Lemminkäisenkatu 14–18, Turku, FI-20520 Finland

**Keywords:** Respiratory, Neonatology, Equine, Horse, Lung, Pulmonary

## Abstract

**Background:**

Pneumonia is a common condition in ailing neonatal foals, and it remains an important cause of morbidity and mortality in this veterinary patient group. Factors affecting the survival of young foals with pneumonia have not been thoroughly investigated. The aim of this study was to explore the potential prognostic factors associated with survival of these foals. Fifty foals under one month of age with pneumonia were included in this retrospective clinical study. The foals were divided into groups based on survival (survived to discharge or died/euthanised during hospitalisation). Multiple clinical and laboratory variables were investigated as risk factors with univariate logistic regression analyses and subsequently with multivariate analyses. If a variable showed prediction potential in regression analysis, a receiver operating characteristic (ROC) analysis was conducted.

**Results:**

In univariate analysis, odds (OR, 95% CI) of non-survival were associated with higher respiratory rate (RR) on the first day after admission to hospital (D1) (1.32, 1.07–1.62, *P* = 0.009, for each 5-unit increase) and positive bacterial blood culture (12.08, 1.88–77.67, *P* = 0.009). Odds of non-survival were decreased for Standardbred breed (0.11, 0.01–0.96, *P* = 0.046) and for foals with longer hospitalisation, with each additional day in hospital further reducing the odds (0.59, 0.40–0.86, *P* = 0.006). In multivariate analysis, odds of non-survival were associated only with higher RR on D1 (1.36, 1.07–1.71, *P* = 0.011, for each 5-unit increase). In ROC analysis, optimal cut-off value for RR was ≥ 55/min with sensitivity 75.0% and specificity 76.3%. Based on predictive values, RR < 55/min on D1 favoured survival.

**Conclusions:**

Higher RR on D1 is a predictor of non-survival in foals with pneumonia in this study, increasing the odds of death by 36% for each 5-unit increase in RR. Respiratory rate below 55/min on D1 favours survival. These findings could assist in early identification of foals that are at increased risk of mortality, thereby aiding in treatment decisions.

**Supplementary Information:**

The online version contains supplementary material available at 10.1186/s12917-024-04405-0.

## Background

The respiratory tract is one of the most important routes of entry for bacteria and other pathogens into a neonatal foal’s body [1]. Therefore, it is hardly surprising that pneumonia is a common disease in young foals [2]. Foals may develop pneumonia as a primary disease, but the likelihood of developing bacterial pneumonia secondary to sepsis or aspiration is higher [2]. Pneumonia as a manifestation of sepsis remains an important cause of morbidity and mortality in neonatal foals. Infection may occur in utero, during parturition or in the perinatal period, and is commonly preceded by failure of passive transfer of immunoglobulins [3]. Besides bacteria, viruses and fungi can contribute to respiratory infection in foals [3]. 

Factors affecting survival of foals with other conditions, such as sepsis [4] and septic arthritis [5], have been investigated, and prognosis for neonatal foals in intensive care assessed [6, 7]. However, pneumonia-related factors have been sparsely evaluated. Failure of transfer of passive immunity has previously emerged as a risk factor for thoracic radiographic changes in foals [8]. Azotaemia, dyspnoea, history of dystocia, and increased anion gap in arterial blood were associated with non-survival in these foals, but physical examination variables, such as fever and tachypnoea, were not [8]. Systemic inflammatory response syndrome (SIRS) was the most common diagnosis of these foals, and pneumonia as a specific diagnosis was not included in the analysis. In intensive care setting, lower respiratory disease is known to increase mortality rate in critically ill foals [9, 10]. Since factors affecting survival need to be better defined in foals with pneumonia, this study aimed to investigate potential prognostic factors associated with their survival.

## Methods

The Viikki Campus Research Ethics Committee of Animal Research at the University of Helsinki approved the study (Approval date 3 November 2020, Approval number 13/2020).

### Animals

Altogether 50 client-owned foals (22 fillies, 28 colts) treated at the Equine Teaching Hospital, University of Helsinki, Finland, during the years 2012–2023 were included in this retrospective clinical study. Inclusion criteria for the foals were age under one month and clinical diagnosis of pneumonia. Pneumonia was diagnosed based on the foal’s history, clinical examination, haematology, serum biochemistry, partial pressure of oxygen in arterial blood (PaO_2_), serum immunoglobulin G (IgG) determination (SNAP Foal IgG test, IDEXX Laboratories Inc., Westbrook, ME, USA), blood culture, thoracic ultrasonography, and/or radiography. Diagnosis was set by the attending clinician specialised in equine medicine (board-certified and/or national specialisation).

### Data acquisition

The medical records of the foals were obtained from an institutional veterinary patient database software (Provet, Nordhealth Finland Oy, Helsinki, Finland). Relevant electronic patient files for this study were identified by searching the database for keywords in the diagnoses, including “pneumonia” and “lung infection,” in both English and Finnish (the native language). All available foals under one month of age with a diagnosis of pneumonia were included, and the study did not have specific exclusion criteria. Data relevant to the study were copied manually onto an Excel worksheet (Microsoft Office Excel 2023, WA, USA).

### Study design

The outcome for the foals was defined by survival (discharged from the hospital alive) or non-survival (died or euthanised during hospitalisation). The reason for death or euthanasia (related to pneumonia, not related to pneumonia, pneumonia and comorbidity, financial, unknown) and comorbidities, diagnosed by the attending clinician, were recorded. The following clinical and laboratory variables were investigated to identify their potential role as risk factors or indicators of survival: sex, age in days, breed, weight, days of gestation of the mare, hospitalisation days, IgG on admission (< 400 mg/dL, 400–800 mg/dL, > 800 mg/dL), bacterial blood culture (positive/negative, bacterial species), body temperature, respiratory rate (RR), heart rate (HR), leucocyte count, and PaO_2_. The variables were recorded daily during the foal’s hospitalisation starting from the day of admission (D0) to the end of hospitalisation (D1, D2, D3, etc.).

### Statistical methods

All statistical analyses were performed using SAS System for Windows, version 9.4 (SAS Institute Inc., Cary, NC, USA). *P*-values were considered significant at < 0.05. Due to the exploratory nature of the study, no corrections for multiplicity were applied. Descriptive statistics and frequency tables were created for potential risk factor variables by survival/non-survival status. Risk factors for non-survival were first assessed with univariate logistic regression analyses. An odds ratio (OR) greater than 1 indicated a positive association with non-survival, whereas a ratio less than 1 indicated a negative association. A multivariate logistic regression analysis was then conducted to control for potentially confounding risk factors for the clinically relevant risk factors with *P*-value < 0.05 in univariate analyses. Odds ratios with 95% confidence intervals (CIs) for non-survival were calculated from the models and illustrated with forest plots. Due to the limited number of non-survival cases, possible interaction effects between the risk factors were not evaluated.

Regarding the variables measured daily (HR, RR, body temperature, leucocytes, PaO_2_), only the measurements at D0, D1, D2, and D3 were considered potential risk factors; further hospitalisation days were excluded from the analysis due to the amount of missing data caused by differing lengths of hospitalisation. In a few cases, the measurement was missing from D1 but measured at D0. In these cases, the missing value on D1 was imputed from the observed measurement on D0. Similarly, missing value on D0 was imputed from observation on D1. No imputations were performed for other days. For sensitivity purposes, data from D0 and D1 were analysed also without the forementioned imputation to confirm the results. PaO_2_ cut-off for statistical analysis was two consecutive measurements of ˃70 mmHg [11]. Below cut-off PaO_2_ was then used as a potential risk factor in the regression analysis.

For clinically relevant variables that showed prediction potential in univariate and multivariate logistic regression analyses, a receiver operating characteristic (ROC) analysis was conducted. Optimal cut-off value was determined based on Youden’s index, maximising the sum of sensitivity and specificity. Sensitivity, specificity, positive predictive value, and negative predictive value together with their 95% CIs were then calculated using the optimal cut-off. For interpretation of the area under the curve (AUC), the following guideline values were used: AUC = 0.5 non-informative, 0.5 < AUC ≤ 0.7 less accurate, 0.7 < AUC ≤ 0.9 moderately accurate, 0.9 < AUC < 1 highly accurate, and AUC = 1 perfect test [12]. The ROC analysis was validated using stratified 5-fold cross-validation, which was repeated two times. Mean prediction accuracy and mean AUCs were calculated for the testing sets.

## Results

Mean age (± SD, range) of the foals on admission was 4 days (± 6.4, 0.5–26 days), and breeds included were Standardbred (*n* = 18), Finnhorse (*n* = 8), Warmblood (*n* = 21), and Pony (*n* = 3). The foals weighed 57.9 kg (± 16.8, 20–114 kg). Thirty-eight foals out of 50 (76%) survived to discharge and 12 foals out of 50 (24%) either died (*n* = 1) or were euthanised (*n* = 11). The reason for non-survival was related to pneumonia (*n* = 3), not related to pneumonia (*n* = 1), or related to pneumonia and comorbidity (*n* = 8). The length of hospitalisation was 7 (±3) days for foals that survived to discharge and 4 (±2) days for foals that died or were euthanised. In Standardbreds, the proportion of survival was the highest (94% [17/18] survived) and in Finnhorses the lowest (50% [4/8] survived). In Warmbloods and Ponies, the proportion of survival was 71% (15/21) and 67% (2/3), respectively. Characteristics and frequency data are presented in Tables 1 and 2 by the survival status of enrolled foals, including frequency data on comorbidities (sepsis, omphalitis, septic arthritis, hypoxic ischaemic encephalopathy, non-infectious orthopaedic conditions, gastrointestinal tract diseases, or other diseases).

**Table 1:** Descriptive statistics and frequency data for investigated variables by survival status in foals with pneumonia

Variable		Survived	Died/Euthanised
Age (days)	Mean (±SD)	3.1 (±4.8)	4.8 (±7.7)
	Range	0.5–21.0	0.5–26.0
	*n*	38	12
Sex, *n* (%)	Filly	18 (47.4)	4 (33.3)
	Colt	20 (52.6)	8 (66.7)
Breed, *n* (%)	Standardbred	17 (44.7)	1 (8.3)
	Finnhorse	4 (10.5)	4 (33.3)
	Warmblood	15 (39.5)	6 (50.0)
	Pony	2 (5.3)	1 (8.3)
Weight (kg)	Mean (±SD)	55.4 (±14.2)	65.9 (±22.4)
	Range	20.0–91.0	39.0–114.0
	*n*	36	11
Gestation duration (days)	Mean (±SD)	335 (±11)	335 (±11)
	Range	317–354	319–350
	*n*	26	7
Hospitalisation duration (days)	Mean (±SD)	7 (±3)	4 (±2)
	Range	2–14	2–8
	*n*	38	12
IgG level on admission, *n* (%)	< 400 mg/dL	11 (33.3)	2 (20.0)
	400–800 mg/dL	2 (6.1)	1 (10.0)
	> 800 mg/dL	20 (60.6)	7 (70.0)
	*n*	33	10
Blood culture, *n* (%)	Positive	6 (17)	5 (71)
	Negative	29 (83)	2 (29)
	*n*	35	7
Comorbidity with pneumonia, *n* (%)	Yes	34 (89.5)	11 (91.7)
	No	4 (10.5)	1 (8.3)
Comorbidity type,^a^* n* (%)			
Sepsis (clinical diagnosis)	Yes	18 (47.4)	4 (33.3)
	No	20 (52.6)	8 (66.7)
Omphalitis	Yes	8 (21.1)	1 (8.3)
	No	30 (78.9)	11 (91.7)
Septic arthritis	Yes	3 (7.9)	1 (8.3)
	No	35 (92.1)	11 (91.7)
HIE	Yes	14 (36.8)	2 (16.7)
	No	24 (63.2)	10 (83.3)
Non-infectious orthopaedic condition^b^	Yes	9 (23.7)	1 (8.3)
	No	29 (76.3)	11 (91.7)
GI disease^c^	Yes	10 (26.3)	2 (16.7)
	No	28 (73.7)	10 (83.3)
Other disease	Yes	8 (21.1)	7 (58.3)
	No	30 (78.9)	5 (41.7)

The total number of foals was 50. The survival status was defined as survived or died/euthanised. SD, standard deviation; IgG, immunoglobulin G; *n*, number of foals with available data; HIE, hypoxic ischaemic encephalopathy; GI, gastrointestinal. The percentages indicate the proportions within groups

^a^Comorbidities encompass the entire hospitalisation period

^b^For example, angular limb deformities or tendon laxity

^c^For example, colic or diarrhoea

**Table 2:** Descriptive statistics and frequency data for investigated continuous variables in foals with pneumonia

Variable	Group		D0	D1	D2	D3
HR	Survived	Mean (±SD) Range	112 (±21) 78–180	103 (±23) 64–170	95 (±18) 68–144	93 (±13) 70–120
		*n*	37	38	35	35
	Died/ Euthanised	Mean (±SD) Range	99 (±33) 55–160	106 (±18) 84–140	108 (±25) 88–160	102 (±19) 78–124
		*n*	11	11	7	6
RR	Survived	Mean (±SD) Range	44 (±15) 20–80	43 (±16) 16–80	41 (±16) 16–80	42 (±21) 12–80
		*n*	34	37	34	33
	Died/ Euthanised	Mean (±SD) Range	51 (±22) 24–90	62 (±24) 26–108	45 (±20) 25–76	46 (±26) 24–84
		*n*	11	11	6	6
Temp (°C)	Survived	Mean (±SD) Range	38.5 (±0.7) 37.0–40.3	38.6 (±0.6) 37.5–40.6	38.5 (±0.3) 38.0–39.0	38.5 (±0.4) 37.9–39.5
		*n*	37	38	36	36
	Died/ Euthanised	Mean (±SD) Range	38.3 (±1.4) 35.3–40.6	38.4 (±1.0) 36.4–39.6	38.2 (±0.6) 37.4–38.8	38.1 (±0.8) 36.7–39.0
		*n*	12	11	7	6
Leuc (x10^9^/L)	Survived	Mean (±SD) Range	9.7 (±10.3) 0.8–61.4	10.6 (±14.0) 1.3–56.8	8.4 (±7.1) 2.8–32.2	9.6 (±6.6) 3.8–32.3
		*n*	34	20	15	19
	Died/ Euthanised	Mean (±SD) Range	8.5 (±6.0) 1.3–19.7	8.6 (±4.3) 3.4–14.7	4.7 (±1.9) 2.9–7.2	5.5 (±2.9) 2.2–7.8
		*n*	11	7	4	3
PaO_2_ (mmHg)	Survived	Mean (±SD) Range	58.1 (±14.6) 28.8–94.9	58.6 (±12.9) 35.3–93.8	60.0 (±11.3) 32.8–82.1	60.5 (±13.4) 36.6–99.2
		*n*	33	33	31	26
	Died/ Euthanised	Mean (±SD) Range	54.7 (±17.0) 36.9–89.1	57.7 (±18.0) 35.9–85.4	55.8 (±12.1) 36.4–65.9	54.9 (±11.9) 41.8–67.3
		*n*	7	7	5	6

The continuous variables were measured daily during hospitalisation of the foals (*N* = 50). Results are presented by survival status (survived and died/euthanised). Data from admission of the foal to hospital (D0) until hospitalisation day 3 are presented (D1–D3). HR, heart rate per minute; RR, respiratory rate per minute; Temp, body temperature; Leuc, leucocyte count; PaO_2_, partial pressure of oxygen in arterial blood; SD, standard deviation; *n*, number of foals with available data

### Univariate logistic regression analysis

**Fig. 1:**
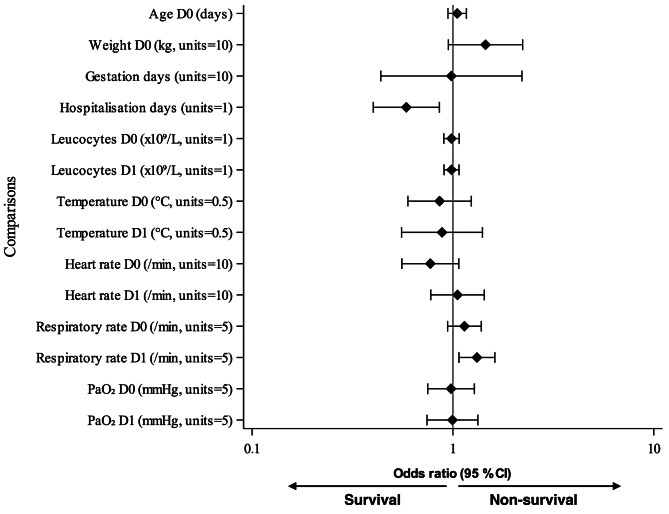
Forest plot of odds ratios of continuous variables related to non-survival prediction in foals with pneumonia (univariate analysis, *N* = 50). Data for D0 (day of admission to hospital) and D1 (first day after admission) are presented. CI, confidence interval; PaO_2_, partial pressure of oxygen in arterial blood

**Fig. 2:**
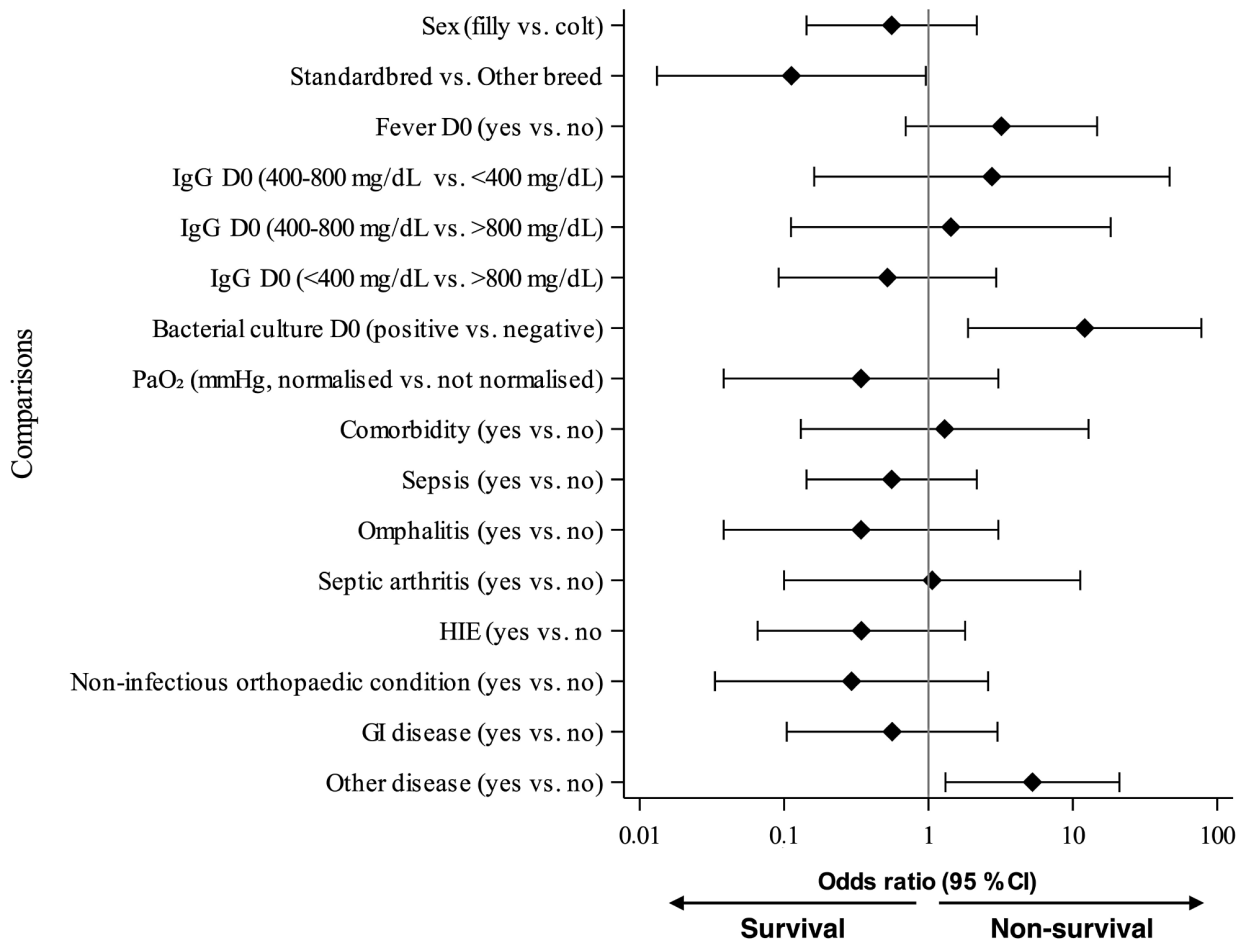
Forest plot of odds ratios of categorical variables related to non-survival prediction in foals with pneumonia (univariate analysis, *N* = 50). Comorbidities encompass the entire hospitalisation period. CI, confidence interval; GI, gastrointestinal; HIE, hypoxic ischaemic encephalopathy; IgG, immunoglobulin G; PaO_2_, partial pressure of oxygen in arterial blood. Non-infectious orthopaedic condition as comorbidity includes, for example, angular limb deformities or tendon laxity, and GI disease includes, for example, colic or diarrhoea

Four variables (higher RR on D1, positive bacterial blood culture, Standardbred breed and longer hospitalisation) reached statistical significance as risk factors for non-survival in univariate analysis (Figs. 1 and 2, Additional file 1). Higher RR on D1 (first day after admission) predicted non-survival with OR of 1.32 (95% CI 1.07–1.62, *P* = 0.009). Thus, the odds for non-survival were increased by 32% for each 5-unit increase in RR on D1. After imputation of D0 and D1 missing data regarding RR (*n* = 5 measurements for D0 and *n* = 2 measurements for D1) was eliminated from the analysis, the result for RR on D1 remained similar (OR 1.30, 95% CI 1.06–1.60, *P* = 0.013). Thus, D0 and D1 results with imputation were used in further analyses regarding RR. A positive bacterial blood culture predicted non-survival with OR of 12.08 (95% CI 1.88–77.67, *P* = 0.009). The cultured bacteria in the non-survival group included *Escherichia coli*, *Streptococcus* sp., *Streptococcus equi* subsp. *zooepidemicus*,* Listeria monocytogenes*, and *Klebsiella pneumoniae* (*n* = 1 for each), and in the survival group *Escherichia coli* (*n* = 4), *Actinobacillus suis* (*n* = 1), and *Staphylococcus aureus* (*n* = 1).

Due to low numbers of Finnhorses and Ponies in the study data and high proportion of survival in Standardbreds, the breeds were dichotomised to Standardbred versus other breeds combined for univariate analysis. The probability for non-survival was decreased (i.e., survival was more likely) for Standardbred breed and for foals with longer hospitalisation, with each additional day in hospital further reducing the odds (OR 0.11, 95% CI 0.01–0.96, *P* = 0.046 and OR 0.59, 95% CI 0.40–0.86, *P* = 0.006, respectively). Having “other disease” (*n* = 7/38 in survived foals, *n* = 7/12 in foals that died or were euthanised) was found to predict non-survival with OR of 5.25 (95% CI 1.31–21.0, *P* = 0.019). None of the other examined comorbidities presented in Table 1 were related to survival. Diagnoses included in “other disease” comprised one of each of the following: bladder rupture, inguinal hernia, septic cellulitis of one limb, incision infection after surgery, congenital polycystic kidney disease, rib fracture, post-operative ileus, pericarditis, dysmaturity, neonatal isoerythrolysis, dorsal displacement of soft palate, and subepiglottic cyst, and two of each of the following: patent urachus, acute renal failure, and corneal ulcer. No association was found between non-survival of the foal and other investigated parameters on hospitalisation days 0–3.

### Multivariate logistic regression analysis

Clinically relevant risk factors with *P*-value < 0.05 were included in multivariate analysis: RR on D1, foal having “other disease” as comorbidity, and Standardbred vs. other breeds. Blood culture (positive/negative) was not included in multivariate analysis due to extent of missing data in the non-survival group. Only RR on D1 retained statistical significance in the multivariate model with similar OR as in the univariate model (OR 1.36, 95% CI 1.07–1.71, *P* = 0.011), indicating 36% increase in odds of non-survival for each 5-unit increase in RR on D1. The variables “other disease” and Standardbred breed showed a trend similar to univariate analyses but without statistical significance in the final multivariate model (OR 4.09, 95% CI 0.78–21.06, *P* = 0.092 for “other disease”; OR 0.12, 95% CI 0.01–1.35, *P* = 0.085 for Standardbred vs. other breeds).

### Receiver operating characteristic analysis

**Fig. 3:**
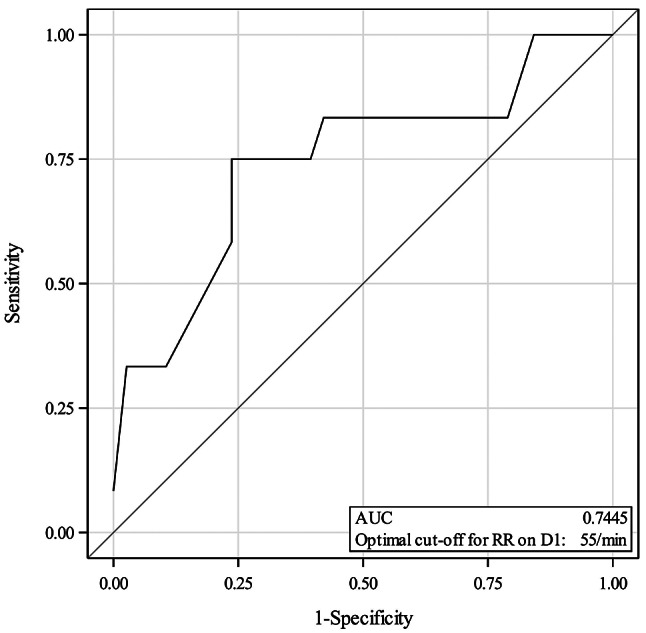
Receiver operating characteristic (ROC) curve for detecting non-survival with respiratory rate (RR) on first day after admission (D1) in foals with pneumonia (*N* = 50)

Receiver operating characteristic analysis was conducted to investigate the prediction potential of RR on D1 for non-survival. Optimal cut-off value for RR based on Youden’s index was ≥ 55/min, which maximised the sum of sensitivity and specificity (Fig. 3). With this cut-off, on D1 the sensitivity of RR was 75.0% (95% CI 50.5–99.5%) and specificity 76.3% (95% CI 62.8–89.8%). The positive predictive value of RR on D1 of ≥ 55/min was 50.0% (95% CI 26.9–73.1%) and negative predictive value 90.6% (95% CI 80.5–100%). The AUC of the ROC was 0.745 (95% CI 0.57–0.92), indicating a moderately accurate test [12]. The cross-validation of the ROC analysis showed a mean accuracy of 81% and a mean AUC of 0.762, supporting the analysis conducted on the full study data.

## Discussion

This study investigated the potential prognostic factors associated with non-survival of young foals diagnosed with pneumonia. We found that foals presenting with higher RR on D1 had 36% increase (in multivariate analysis) in odds for non-survival. Foals that died or were euthanised presented on D1 with a RR of 19/min (± 8/min) higher than foals that survived. The cause of higher RR on D1 in foals that did not survive can be speculated. Tachypnoea is commonly secondary to primary lung disease, such as pneumonia [13], which could be the case here. However, tachypnoea can also be present in several other conditions, e.g., as a physiological response or increased sympathetic drive (fever, fear, pain, excitement), metabolic and respiratory acidosis, other pulmonary pathologies such as meconium aspiration, pleural effusion, or surfactant deficiency in premature/dysmature foals, lung contusion due to rib fracture, central nervous system dysfunction, or cardiovascular diseases [13, 14]. The combined effect of these contributing factors might account for some cases of increased RR, and the foals diagnosed with pneumonia often had several comorbidities. A rapid response to treatment might explain the difference between groups for RR on D1; foals responding well to antimicrobial and other therapy initiated on admission day could have had lower RR the next day, unlike foals with poorer response to treatment and eventual non-survival. However, other factors, such as normalised body temperature and corrected acid-base balance, could also explain the lowered RR in foals that survived.

In children, tachypnoea is often used as one of the major clinical markers for pneumonia [15, 16], although studies regarding its predictor potential for pneumonia have yielded conflicting results [17–19]. Furthermore, increased mortality rate associated with tachypnoea as a marker of respiratory distress has been reported in children [20–23]. Our study provides novel information on the survival prediction potential of tachypnoea in young foals with pneumonia. This finding contrasts with the results of Bedenice et al., [8] who found that in their study of foals with radiographic evidence of lower airway disease, physical examination findings, such as tachypnoea, were not significant prognostic factors for outcome. However, their study was not specific to pneumonia, and the study design differed with a focus on thoracic radiographic changes, and therefore their results are not fully comparable to ours.

The optimal cut-off for RR on D1 was 55/min in ROC analysis, which is clearly higher than the normal respiratory rate in young foals (20–40/min for foals over 12 h of age) [24]. Age affects RR, and foals under 12 h of age may have higher RR, but the youngest foals included in this study were 12 h old. When looking at the prediction potential of RR on D1 for non-survival with the cut-off of 55/min, the sensitivity and specificity were 75% and 76%, respectively. For a test to be clinically useful, the sum of sensitivity and specificity should be at least 1.5 [25], which is the case here (0.75 + 0.76 = 1.51). However, these results should be interpreted with caution considering the wide confidence intervals, and further statistical testing preferably with larger sample size should be conducted to confirm the results.

On D1, the positive predictive value of RR was lower (50%) than the negative predictive value (90%) in our study. Only 50% of foals with RR over 55/min died or were euthanised at the end of their hospital treatment, and 90% of the foals with RR below 55/min survived. Thus, having RR lower than 55/min on D1 seems to reliably predict survival in these foals, but RR over 55/min is less useful regarding survival. One of the possible factors affecting this finding might be the comorbidities of the foal. It is noteworthy that the prevalence of the investigated condition (non-survival in this case) affects predictive values; the lower the probability of a condition, the lower the predictive values [25]. The numbers of foals in our study groups were not equal, as 38/50 foals (76%) survived to discharge, while 12/50 foals (24%) either died or were euthanised, indicating a clearly lower prevalence for non-survival. Our finding regarding the predictive potential of RR on D1 for non-survival should be confirmed with a larger sample size and with more equally sized groups.

Positive bacterial blood culture showed high odds for non-survival in our study. This finding contrasts with those of Bedenice et al. (2003) [8] but is consistent with the findings of a large study on foals treated in a neonatal intensive care unit during 1982–2008 [9]. Additionally, Kumar et al. [26] found positive blood culture to be associated with mortality in neonatal babies with pneumonia. However, given the wide confidence intervals for odds in the current study and the fact that the number of foals that died or were euthanised with a bacterial blood culture performed was low (7/12 of which 5/7 [71%] were positive), our results should be interpreted with caution. In foals that survived, blood culture was positive only for 6/35 foals (17%). The number of each cultured bacterial species was low in both groups, and therefore the potential effect of certain species on survival could not be investigated.

The duration of hospitalisation was longer for foals that survived (Table 1; Fig. 1), indicating that death or euthanasia could have been due to lack of response to treatment, and thus, shorter hospitalisation. However, financial impact on the decision to euthanise a foal cannot be excluded, although none of the foals’ medical records mentioned this as the primary reason. Euthanasia for financial reasons could have led to misclassification of potential survivors as non-survivors, but the potential effect of this bias on the results remains unclear. While longer hospital treatment appears to favour survival, comorbidities can also have a marked effect on the length of hospitalisation. For ethical reasons and to diminish the high costs of treatment, the prognosis for survival must be determined as early as possible and consider all conditions of the foal. This enables owners to make informed decisions about management options for the foal.

Finally, compared to other breeds, Standardbred foals had the highest survival rate in this population. The reason for this is unknown and may be coincidence, but it could also potentially indicate that Standardbred foals are more valuable to their owners, leading to earlier admission to hospital and greater investment and commitment to treatment. If cared for by experienced professionals (such as stud farm personnel) rather than private owners, foals’ clinical signs could be detected sooner, allowing for earlier hospitalisation and/or initiation of treatment. This could contribute to the high survival rate. These aspects could not be investigated further from these data, and it remains unclear whether factors related to Standardbred breed per se influenced survival.

Some limitations of this study should be addressed. The study was retrospective, and we relied on the precision and thoroughness of medical records of the foals and the accuracy of the pneumonia and comorbidity diagnoses set by the attending clinicians. However, not all records were detailed and some had missing data. For example, the sepsis score was not available for all foals and was therefore omitted from the analysis. Nevertheless, it is likely that the attending clinicians’ judgment at the time of the foals’ treatment was more accurate than any retrospective reclassification based on medical record review. The number of foals in this retrospective study was relatively low, which can affect the statistical analysis. The results should be confirmed with larger numbers of foals. Another limitation of this study was that no tracheal wash was obtained to confirm pneumonia. Although this would be ideal, it is not routinely performed for young foals due to the invasive nature and costs of the procedure. The reasons for pneumonia (bacterial, viral, fungal, meconium or milk aspiration, etc.) were not investigated separately, and therefore the different etiological agents and their contribution to survival could not be evaluated. Some clinical variables used to diagnose pneumonia, such as auscultation findings, are subjective and were therefore omitted from this study. Ultrasonography and radiography findings were used by attending veterinarians to set the diagnosis. However, for the purposes of this study, imaging findings were neither collected nor categorised by severity. Although PaO_2_ as a prognostic factor for non-survival was not significant in this study, using a cut-off of 70 mmHg for PaO_2_ might have underestimated hypoxaemia in older foals [11]. Most foals had one or more comorbidities with pneumonia, which most likely contributed to the outcome of these foals. Indeed, having “other disease” as a comorbidity, including miscellaneous diagnoses, increased the odds of non-survival in our study. There was a low number of cases for each diagnosis within the variable “other disease” in our studied population, which prevented further interpretation of these findings. Frequently, foals experience multiple disease processes or present with several infection foci simultaneously, often complicating treatment [27]. These collective conditions may increase risk of death and make it a challenge to assess the impact of specific clinical parameters on a single diagnosis.

## Conclusions

We found that higher RR on the first day after admission to hospital is a predictor of non-survival in foals with pneumonia, increasing the odds of death by 36% for each 5-unit increase in RR. Respiratory rate below 55/min on the first day after admission to hospital favours survival. These findings may help in identifying the foals early during hospitalisation that are at higher risk of not surviving. This may aid in treatment decisions. Positive bacterial blood culture and Standardbred breed may also affect the outcome of foals with pneumonia, but these findings warrant further investigation.

## Electronic supplementary material

Below is the link to the electronic supplementary material.

**Supplementary Material 1:** Univariate logistic regression analysis results regarding non-survival prediction in foals with pneumonia (*N*=50)

## Data Availability

The datasets generated and/or analysed during the current study are not publicly available due to reasons of confidentiality (patient data) but are available from the corresponding author on reasonable request.

## Abbreviations

AUC: Area under the curve
CI: Confidence interval
D0: Day of admission to hospital
D1: First hospitalisation day after admission
GI: Gastrointestinal
HIE: Hypoxic ischaemic encephalopathy
HR: Heart rate per minute
IgG: Immunoglobulin G
Leuc: Leucocyte count
OR: Odds ratio
PaO_2_: Partial pressure of oxygen in arterial blood
ROC: Receiver operating characteristic
RR: Respiratory rate per minute
SIRS: Systemic inflammatory response syndrome
Temp: Body temperature
