# Supplementary material for: Factors affecting survival of foals with pneumonia in a referral hospital
Source: BMC Vet Res. 2024 Dec 18;20:562. doi: 10.1186/s12917-024-04405-0 (PMC11653636; doi:10.1186/s12917-024-04405-0)
Supplement: Supplementary file 1 — Supplementary Material 1: Univariate logistic regression analysis results regarding non-survival prediction in foals with pneumonia (N=50) [file 12917_2024_4405_MOESM1_ESM.pdf]

Additional file 1. Univariate logistic regression analysis results regarding non-survival prediction in foals with pneumonia (N=50).

Variable	Odds ratio	Lower CI	Upper CI	P-value
Age D0 (days)	1.050	0.944	1.167	0.3684
Bacterial blood culture D0 (positive vs. negative)	12.083	1.880	77.665	0.0087
Comorbidity (yes vs. no)	1.294	0.130	12.834	0.8257
Fever D0 (yes vs. no)	3.200	0.696	14.720	0.1352
GI disease (yes vs. no)	0.560	0.104	3.008	0.4990
Gestation days (units=10)	0.982	0.437	2.206	0.9650
Heart rate D0 (/min, units=10)	0.765	0.552	1.061	0.1081
Heart rate D0 (/min, units=10, imputed)	0.771	0.557	1.069	0.1187
Heart rate D1 (/min, units=10)	1.053	0.775	1.431	0.7399
Heart rate D2 (/min, units=10	1.371	0.926	2.030	0.1149
Heart rate D3 (/min, units=10)	1.548	0.834	2.875	0.1663
HIE (yes vs. no)	0.343	0.066	1.794	0.2049
Hospitalisation days (units=1)	0.586	0.401	0.856	0.0057
IgG (400–800 mg/dL vs. <400 mg/dL)	2.750	0.162	46.792	0.4842
IgG (400–800 mg/dL vs. >800 mg/dL)	1.429	0.112	18.298	0.7840
IgG (<400 mg/dL vs. >800 mg/dL)	0.519	0.092	2.945	0.4594
Leucocytes D0 (x10^9/L, units=1)	0.983	0.901	1.074	0.7083
Leucocytes D1 (x10^9/L, units=1)	0.984	0.903	1.072	0.7097
Leucocytes D2 (x10^9/L, units=1)	0.705	0.399	1.246	0.2290
Leucocytes D3 (x10^9/L, units=1)	0.695	0.404	1.194	0.1874
Non-infectious orthopaedic condition (yes vs. no)	0.293	0.033	2.590	0.2696
Omphalitis (yes vs. no)	0.341	0.038	3.048	0.3357
Other disease (yes vs. no)	5.250	1.311	21.026	0.0192
PaO_2 D0 (mmHg, units=5)	0.919	0.686	1.233	0.5748
PaO_2 D0 (mmHg, units=5, imputed)	0.978	0.749	1.278	0.8724
PaO_2 D1 (mmHg, units=5)	0.974	0.717	1.324	0.8674
PaO_2 D1 (mmHg, units=5, imputed)	0.994	0.743	1.330	0.9676
PaO_2 D2 (mmHg, units=5)	0.843	0.548	1.298	0.4388
PaO_2 D3 (mmHg, units=5)	0.826	0.556	1.226	0.3421
PaO_2 (mmHg, normalised vs. not normalised)	0.341	0.038	3.048	0.3357
Respiratory rate D0 (/min, units=5)	1.120	0.918	1.366	0.2631
Respiratory rate D0 (/min, units=5, imputed)	1.141	0.941	1.383	0.1802
Respiratory rate D1 (/min, units=5)	1.300	1.058	1.599	0.0126
Respiratory rate D1 (/min, units=5, imputed)	1.317	1.072	1.617	0.0087
Respiratory rate D2 (/min, units=5)	1.076	0.834	1.389	0.5715
Respiratory rate D3 (/min, units=5)	1.047	0.855	1.281	0.6569
Sepsis (yes vs. no)	0.556	0.143	2.162	0.3965
Septic arthritis (yes vs. no)	1.061	0.100	11.260	0.9611
Sex (filly vs. colt)	0.556	0.143	2.162	0.3965

Standardbred vs. Other breed	0.112	0.013	0.959	0.0457
Temperature D0 (°C, units=0.5)	0.861	0.600	1.236	0.4175
Temperature D0 (°C, units=0.5, imputed)	0.858	0.596	1.234	0.4079
Temperature D1 (°C, units=0.5)	0.830	0.514	1.340	0.4451
Temperature D1 (°C, units=0.5, imputed)	0.882	0.555	1.403	0.5965
Temperature D2 (°C, units=0.5)	0.375	0.119	1.184	0.0945
Temperature D3 (°C, units=0.5)	0.375	0.131	1.072	0.0673
Weight D0 (kg, units=10)	1.452	0.948	2.224	0.0860

All investigated variables related to non-survival prediction in foals with pneumonia are presented in alphabetic order. The table includes data from D0 (day of admission to hospital) to D3 (third day after admission day). Comorbidities encompass the entire hospitalisation period, otherwise the hospitalisation day is indicated. CI, confidence interval; PaO_2, partial pressure of oxygen in arterial blood; GI, gastrointestinal; HIE, hypoxic ischaemic encephalopathy; IgG, immunoglobulin G; PaO_2, partial pressure of oxygen in arterial blood. Non-infectious orthopaedic condition as comorbidity includes, for example, angular limb deformities or tendon laxity, and GI disease includes, for example, colic or diarrhoea.
